# Genome‐wide DNA methylation profiling and identification of potential pan‐cancer and tumor‐specific biomarkers

**DOI:** 10.1002/1878-0261.13176

**Published:** 2022-01-21

**Authors:** Joe Ibrahim, Ken Op de Beeck, Erik Fransen, Marc Peeters, Guy Van Camp

**Affiliations:** ^1^ Center of Medical Genetics University of Antwerp and Antwerp University Hospital Edegem Belgium; ^2^ Center for Oncological Research University of Antwerp and Antwerp University Hospital Edegem Belgium; ^3^ StatUa Center for Statistics University of Antwerp Belgium; ^4^ Department of Medical Oncology Antwerp University Hospital Edegem Belgium

**Keywords:** biomarker, cancer, genome‐wide analysis, methylation, pan‐cancer, tumor‐specific

## Abstract

DNA methylation alterations have already been linked to cancer, and their usefulness for therapy and diagnosis has encouraged research into the human epigenome. Several biomarker studies have focused on identifying cancer types individually, yet common cancer and multicancer markers are still underexplored. We used The Cancer Genome Atlas (TCGA) to investigate genome‐wide methylation profiles of 14 different cancer types and developed a three‐step computational approach to select candidate biomarker CpG sites. In total, 1991 pan‐cancer and between 75 and 1803 cancer‐specific differentially methylated CpG sites were discovered. Differentially methylated blocks and regions were also discovered for the first time on such a large scale. Through a three‐step computational approach, a combination of four pan‐cancer CpG markers was identified from these sites and externally validated (AUC = 0.90), maintaining comparable performance across tumor stages. Additionally, 20 tumor‐specific CpG markers were identified and made up the final type‐specific prediction model, which could accurately differentiate tumor types (AUC = 0.87–0.99). Our study highlights the power of the methylome as a rich source of cancer biomarkers, and the signatures we identified provide a new resource for understanding cancer mechanisms on the wider genomic scale with strong applicability in the context of new minimally invasive cancer detection assays.

## Graphical abstract

We used The Cancer Genome Atlas to study genome‐wide methylation profiles of 14 different cancer types. We identified and validated a combination of four candidate pan‐cancer methylation CpG biomarkers and 20 candidate cancer type‐specific markers. This highlights the methylome as a rich source of biomarkers which can be used in the context of new minimally invasive cancer detection assays.

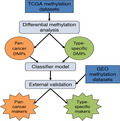

**Abbreviations**
AIC: Akaike information criterion
AUC: area under the curve
BLCA: bladder urothelial carcinoma
BRCA: breast carcinoma
cfDNA: cell‐free DNA
CRC: colorectal carcinoma
ctDNA: circulating tumor DNA
DMB: differentially methylated blocks
DMP: differentially methylated probe
DMR: differentially methylated regions
DNA: deoxyribonucleic acid
ESCA: esophageal carcinoma
GEO: gene expression omnibus
HNSC: head and neck squamous cell carcinoma
KIRC: kidney renal clear cell carcinoma
KIRP: kidney renal papillary cell carcinoma
LIHC: liver hepatocellular carcinoma
LUAD: lung adenocarcinoma
LUSC: lung squamous cell carcinoma
MHC: major histocompatibility complex
PAAD: pancreatic adenocarcinoma
PCR: polymerase chain reaction
PLSDA: partial least squares‐discriminant analysis
PRAD: prostate adenocarcinoma
ROC: receiver operating characteristic
SNP: single nucleotide polymorphism
TCGA: The Cancer Genome Atlas
THCA: thyroid carcinoma
UCEC: uterine corpus endometrial carcinoma
ΔβFC: Δβ fold change

## 1 Introduction

Cancer is the second leading cause of mortality worldwide, with breast, prostate, lung, colorectal, and gastric tumors being the most incident [1]. Abnormal DNA methylation is considered a hallmark of cancer development and where both a global hypomethylation and a locus‐specific hypermethylation have been observed [2]. Tumor cells exhibit a global loss of methylation in otherwise extensively methylated regions (repeat elements, satellites, and retrotransposons), which leads to widespread genomic instability and oncogene activation. Contrastingly, hypermethylation at specific loci is usually observed at promoter CpG islands of tumor suppressor genes, which leads to their repression and transcriptional silencing [3]. Such a phenomenon is often not limited to certain CpG islands, but occurs at multiple independent genomic loci. This is indicative of a widespread deregulation in DNA methylation patterns in the different tumor types [4].

Given that DNA methylation plays a pivotal role in cancer, several studies have outlined the use of methylated DNA loci as cancer detection markers, focusing mainly on gene promoter markers or single CpG markers [5, 6]. Although several such methylation biomarkers have been identified, only a few of them have been used in the clinic. These assays are still plagued by inconsistent performance across cancer stages and inadequacy for detecting residual disease [5, 7]. Moreover, existing biomarkers still mostly target one or a couple cancer types in their mode of action. Robust biomarkers that can detect or diagnose cancer, based on shared methylation patterns between the different cancer types [8], termed pan‐cancer biomarkers, are yet to be described. Some pan‐cancer differential methylation patterns have already been studied, but have not yet been used in the context of biomarkers [9, 10]. Contrastingly, effective multicancer tumor‐specific methylation markers are also still understudied. Most DNA methylation profiling studies have traditionally focused on gene promoter regions and CpG islands, although methylation alterations in non‐island loci including gene bodies and shore regions have also been shown to regulate gene expression [11]. Additionally, cancer tissue‐specific gene expression patterns have been linked to differences in DNA methylation patterns in shore and gene body regions [12].

In this study, we combined genome‐wide differential methylation profiling at a single CpG site resolution, with machine learning to identify both pan‐cancer and type‐specific detection markers. Our analysis was performed using publicly available data from The Cancer Genome Atlas (TCGA), one of the largest cancer patient methylation datasets (*N* = 6502). We present a selection of highly informative CpG sites, identified genome‐wide, that could function as pan‐cancer detection markers as well as a selection of tumor‐specific markers. We also outline comprehensive differential methylation profiles across cancer types which highlight the methylome as an abundant source of biomarkers and its applicability in translational approach for the diagnosis and treatment of cancer.

## 2 Materials and methods

### 2.1 Datasets and study population

DNA methylation datasets were downloaded from the TCGA Data Portal using an in‐house developed Python script as described in Ibrahim et al. [13]. TCGA includes methylation data for more than 30 different tumor types, but some of these sets have a low case to control ratio or lack control samples altogether, preventing robust statistical analyses. In accordance with other studies on TCGA data, we only chose datasets that had a tumor‐to‐normal sample ratio of 10% or a minimum of 10 tumor‐normal pairs [14, 15]. In total, datasets for 14 distinct tumor types were used for analysis (colon and rectal tumor datasets were combined to form the colorectal cancer dataset). The final tally comprised 6502 samples in total, 5783 cases and 719 controls. Biospecimen and clinicopathological data for the different cancer types were similarly downloaded from the portal. Table 1 presents an overview of the described TCGA datasets. For independent external validation, 10 additional Illumina 450K methylation datasets were downloaded from the Gene Expression Omnibus (GEO) database. These were aggregated into one larger set comprising 332 normal and 1263 tumor samples across eight different tumor types to represent a pan‐cancer validation set (Table S1).

**Table 1:** Overview of the TCGA datasets and their clinicopathological parameters used in the analysis. NT, normal tissue; SD, standard deviation; TP, primary tumor.

Dataset name (TCGA abbreviation)	#NT	#TP	#Total	Gender (M/F)	Mean age	Clinical stage (I/II/III/IV)
Bladder urothelial carcinoma (BLCA)	21	418	439	304/108^a^	68.10	2/131/141/136^b^
Breast carcinoma (BRCA)	96	791	887	9/780^a^	58.09	127/443/199/11^b^
Colorectal carcinoma (CRC)	45	411	456	211/179^a^	64.46	55/144/119/54^b^
Esophageal carcinoma (ESCA)	16	185	201	158/27^a^	62.45	19/79/56/9^b^
Head and Neck squamous cell carcinoma (HNSC)	50	528	578	386/142^a^	60.91	27/77/82/270^b^
Kidney renal clear cell carcinoma (KIRC)	160	324	484	205/114^a^	61.37	155/31/73/59^b^
Kidney renal papillary cell carcinoma (KIRP)	45	275	320	202/73^a^	61.68	168/18/51/14^b^
Liver hepatocellular carcinoma (LIHC)	50	377	427	255/122^a^	59.45	175/87/86/5^b^
Lung adenocarcinoma (LUAD)	32	473	505	215/246^a^	65.12	250/113/73/20^b^
Lung squamous cell carcinoma (LUSC)	42	370	412	276/96^a^	67.54	174/135/56/4^b^
Pancreatic adenocarcinoma (PAAD)	10	184	194	102/82^a^	64.76	21/151/5/5^b^
Prostate adenocarcinoma (PRAD)	50	502	552	498/0^a^	61.01	^b^
Thyroid carcinoma (THCA)	56	507	563	136/371^a^	47.26	285/52/113/55^b^
Uterine Corpus Endometrial Carcinoma (UCEC)	46	438	484	0/432^a^	64.18	243/43/101/45^b^
Total	719	5783	6502	2957/2772^a^	61.89	1701/1504/1155/687^b^

^a^ Number not including multiple samples from the same patient.

^b^ Field with missing values.

### 2.2 Methylation data preprocessing

**Fig. 1:**
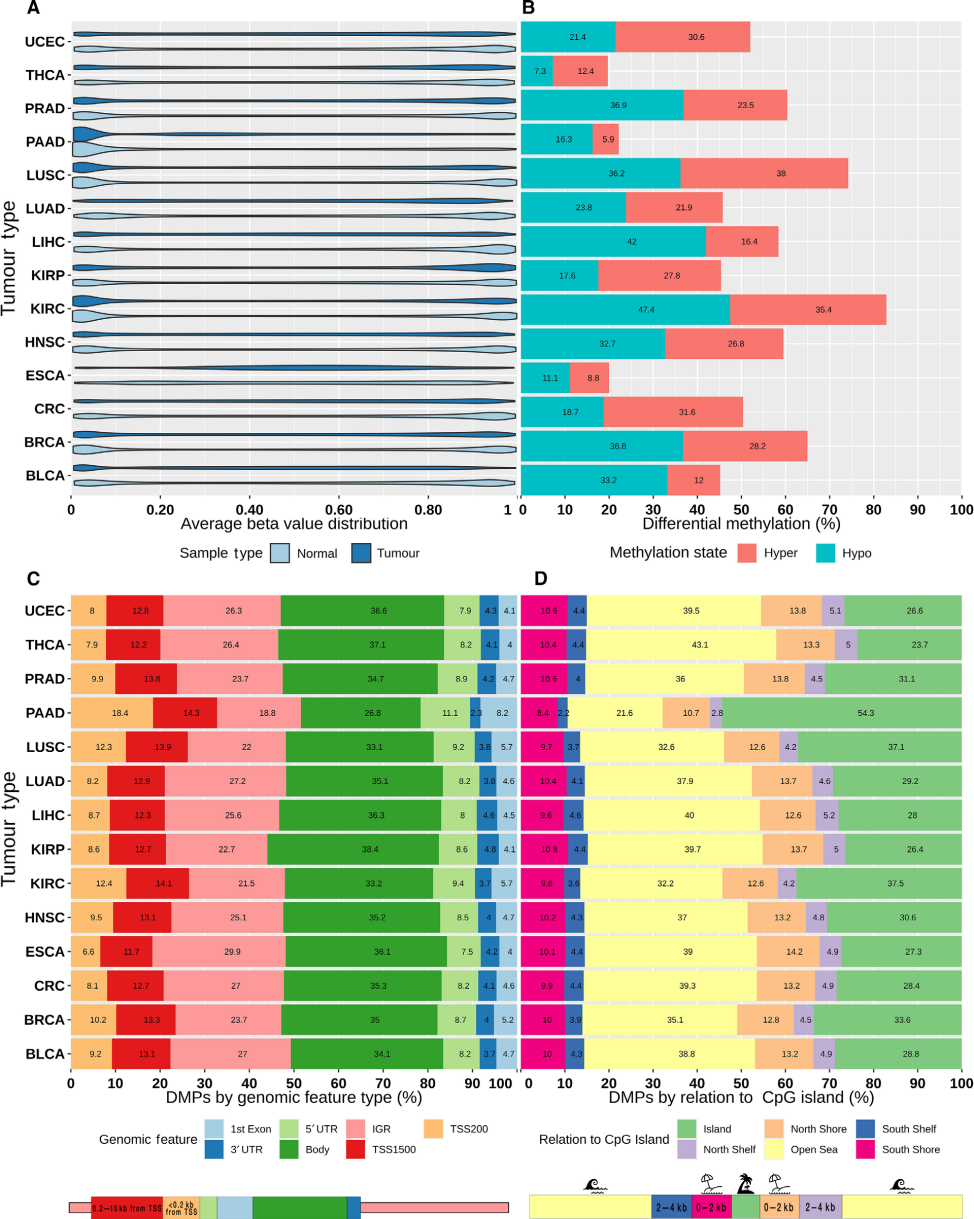
Overview of the differential methylation analysis results across different cancer types. (A) Violin plot of the beta‐value distribution in normal and tumor samples. (B) Bar plot showing the proportion of differentially methylated probes (DMPs) stratified into hyper‐ and hypomethylation. (C) Bar plot showing the proportion of DMPs based on annotated genomic features. (D) Bar plot showing the proportion of DMPs based on their genomic relation to CpG islands. IGR, intergenomic region; TSS, transcription start site; UTR, untranslated region.

Methylation dataset preprocess was performed based on the methods previously described in Ibrahim et al. [13, 16]. Sample methylation data in TCGA were obtained using Illumina’s Infinium HumanMethylation450 BeadChip array, which contains more than 450 000 methylation sites covering 99% of the RefSeq genes. These data come from frozen/formalin‐fixed paraffin‐embedded, resection tissue samples, containing a minimum of 60% tumor nuclei and derived from primary or untreated tumor tissue. In addition to genomic location and other details, the Illumina 450K array manifest denotes probes based on their relation to CpG islands and the type of genomic feature they belong to; these annotations are visualized in Fig. 1. Methylation is reported as β‐value, which is the ratio of the methylated probe intensity over the sum of methylated and unmethylated probe intensities, ranging from 0 for unmethylated to 1 for fully methylated. The downloaded methylation datasets were level 3, meaning that they have already been aggregated, normalized, and/or segmented. Potential batch effects were tested using singular value decomposition, but the data did not present significant effects, which is in line with previous studies using TCGA datasets. To account for the non‐independence between measurements from the same individuals, a linear mixed model was fitted, which included a random effect for sample barcodes while the significance of the fixed effects was tested via the F‐test with a Kenward‐Roger correction for the number of degrees of freedom.

### 2.3 Differential methylation analysis

Differential methylation analysis was mainly carried out using the package *champ* (version 2.12.2) [17]. After data read‐in, samples with more than 25% of their probed data missing were excluded, remaining probes with missing values were filtered and β values less than 0 were set at 0 and values > 1 were set at 1. Underperforming probes were filtered from the downstream analysis; this included control probes, X‐/Y‐chromosome probes, multihit probes, and probes with known single nucleotide polymorphisms (SNPs). We defined Δβ fold change (ΔβFC) for each probe, as the fold change difference in mean β‐value for that probe, between group A and group B (i.e., tumor and normal samples). The criteria for assigning pan‐cancer and tumor‐specific differentially methylated probes (DMPs) for use in the subsequent biomarker analysis were: a log |ΔβFC| ≥ 2 and a corrected *P*‐value ≤ 0.01. *P*‐values were adjusted for multiple testing using the Benjamini–Hochberg correction. Differentially methylated regions (DMRs) and differentially methylated blocks (DMBs), which are extended regions of the genome that exhibit a quantifiable difference in methylation between two groups, were identified using an implemented extension of the *Bumphunter* algorithm in *ChAMP*, with minimum sizes of 50 and 500 bp, respectively.

### 2.4 Pan‐cancer biomarker identification

**Fig. 2:**
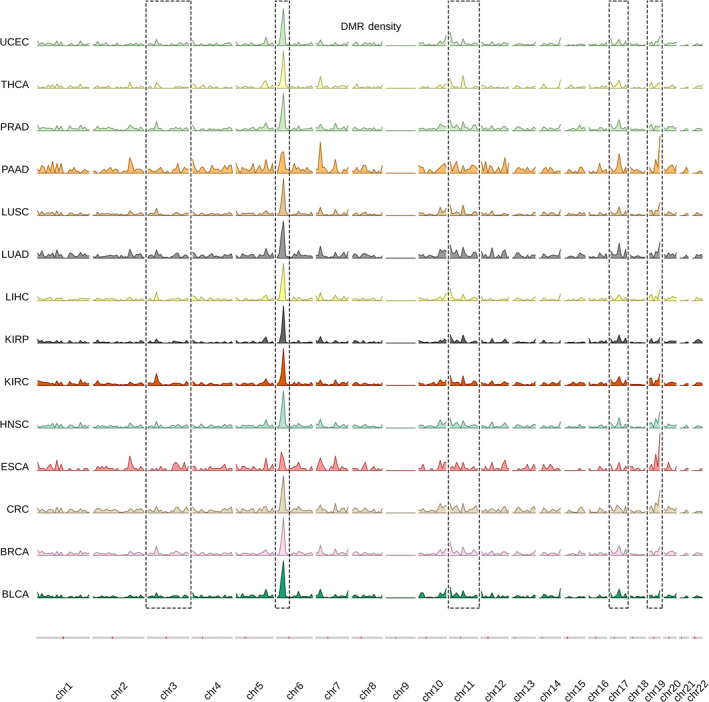
Schematic diagram outlining the biomarker identification methods. Following initial differential methylation analysis and filtering, common differentially methylated probes (DMPs) for all tumor types are considered for pan‐cancer marker identification, while tumor‐specific DMPs are considered for type‐specific marker identification. For pan‐cancer biomarkers, binary logistic regression was used to test classifier models using combinations of four CpG probes as predictors; the final model is then selected based on the best model metrics. This is followed by external validation and stage stratification. For type‐specific biomarkers, a more robust, 3‐step approach was implemented to select the most informative predictors from the initial pool of type‐specific DMPs. The best performing combinations of six CpG probes for each of the 14 cancer types are ultimately selected and integrated in the final classifier model. FDR, false discovery rate; NT, normal tissue; PLSDA, partial least squares‐discriminant analysis; TP, primary tumor.

After the genome‐wide prescreening to identify sites with the most differential methylation between tumor and normal samples was carried out on the individual cancer datasets, DMPs that were common for all 14 types and had a log |ΔβFC| ≥ 2 were selected for the subsequent classifier model building. Combinations of 1, 2, 3, or 4 predictors were tested (Fig. 2), with 4 predictors yielding the best prediction metrics. To select the final model, binary logistic regression models were fitted to predict tissue type (normal/tumor) using different combinations of 4 CpG methylation values as predictors, with a total of 24 157 combinations tested. The final model was chosen based on the highest Akaike information criterion (AIC) and the highest area under the curve (AUC) values. Model prediction accuracy was assessed by plotting receiver operating characteristic (ROC) curves. The final model was then validated in the aggregated GEO external dataset and its performance was plotted. To test the uniformity of the relationship between probe methylation and sample type across stages, we set tissue type (normal or tumor) as a dependent variable and included CpG methylation, stage, and the interaction between methylation and stage, as independent variables in the final regression model. We then tested the significance of the interaction terms using a likelihood ratio test, comparing the fit of the model with both, main effects and their interaction term against the model, with only the main effects of methylation and stage. The final model was then stratified per clinical cancer stage (stage I–IV) and performance recalculated for each of the stages.

### 2.5 Type‐specific biomarker identification

In a similar prescreening setting, a 1‐vs‐all approach was employed to identify differentially methylated probes between 1 of the 14 tumor types and the other 13 combined, using a pooled dataset of 5783 cases comprising the 14 tumor types. DMPs that are specific to each of the tumor types individually, which would serve as model predictors, were identified using a multiclass matrix intersection approach (Venn diagram‐like) and then filtered based on the log |ΔβFC| ≥ 2 criterion. We then used a 3‐step approach to reduce the number of predictors, while retaining the most informative ones and building the multiclass prediction model (Fig. 2). In a first step, we used a modified version of the *Relief* feature selection algorithm, *ReliefF*, to select the most relevant predictors for multiclass classification. In a second step, we implemented a redundancy filter through *K*‐means clustering predictors, to filter out similar features and further minimize the feature set. A total of 10 feature clusters were discovered. From each cluster, two predictors were selected; the one closest to the cluster centroid and the one with the highest Relief score. The third step was model‐building, where we used the partial least squares‐discriminant analysis (PLSDA) algorithm for our multiclass classification of the tissue of origin. To that end, the 14 datasets were pooled together, resulting in a pooled dataset of 5783 tumors each belonging to 1 of the 14 tumor types. The algorithm was run using combinations of six probes from the set of 20 nonredundant predictors. A total of 38 760 combinations were tested and ROC curves with AUC values were generated for predicting each cancer type against the 13 others. The final model was built by integrating the best performing CpG combinations for predicting each of the cancer types. The final model was then externally validated in 10 external methylation datasets downloaded from the GEO covering 8 out of the 14 cancer types (Table S1).

### 2.6 Statistical analyses

The statistical software r (version 3.6.2) [18] was used for all analyses and visualizations. The following clinicopathological parameters from the TCGA clinical patient data files were designated to perform association analyses: age at diagnosis, gender, pathological tumor stage (I–IV). In all regression models, age and tumor stage were accounted for as a covariate, but were excluded from the final model if their effect on the outcome was not significant. Unless stated otherwise, all reported *P*‐values are two‐sided, and those ≤ 0.05 were considered statistically significant. All genomic annotations were done using the GRCh37/hg19 genome build. A complete list of the R packages used can be found on the last page of the supplementary materials document.

## 3 Results

### 3.1 Patterns of differential DNA methylation across tumor types

Most tumor types exhibited overall bimodal β‐value density distributions in both normal and cancer samples, with slightly higher low‐end densities. Esophageal cancer showed a consolidation of β‐values in the midrange for both groups, while pancreatic cancer showed a consolidation almost exclusively at the low end of the β‐value range (Fig. 1A). To allow for a scalable comparison between different tumors (Table 1), DMP counts are reported as normalized proportions based on the total number of analyzed CpGs probes in each category. Differential methylation was significantly variable among the tumor types (Figs S1 and S2 and Tables S2 and S3); on average, 55% differential methylation was observed across the tumor types with 30% hypomethylation and 25% hypermethylation (Fig. 1B). Esophageal, pancreatic, and thyroid cancers had the lowest proportion of differential methylation at around 22% while kidney, lung, and prostate cancers had the highest proportion at 70% or higher. The most hypomethylation was observed in liver, lung squamous cell, and kidney cancers at around 40–48%, while the least hypomethylation was observed in thyroid, esophageal, and pancreatic cancer at around 10–15%. Conversely, breast, lung squamous cell, and kidney cancers had the highest proportion of hypermethylation at 35–39% while bladder, esophageal, and pancreatic cancers had the lowest proportion of hypermethylation at 9–15%. Interestingly, differential methylation was split almost equally between hyper‐ and hypomethylation in breast cancer (Fig. 1B). No significant correlation was observed between the number of DMPs and the number of samples in the datasets (Pearson’s *P*‐value > 0.05). DMPs across the tumor types were mapped to 17 000 unique genes on average, with four DMPs per gene being the most common and an average of eight DMPs per gene (Table S2). On average, 35% of DMPs were located in the gene body, 24% in the IGR, 13% in the TSS1500, 10% in the TSS200, 9% in the 5′ UTR, and 4% in the 3′UTR and 1st exon each (Fig. 1C). With respect to DMP distribution by relation to CpG islands, the largest proportion of DMPs mapped to open‐sea regions at 37% on average followed by CpG islands at 31% on average. North and south shores contained an average of 13% and 10% of DMPs, respectively, while north and south shelves contained the lowest average proportion of DMPs at 5% and 4%, respectively (Fig. 1D).

**Fig. 3:**
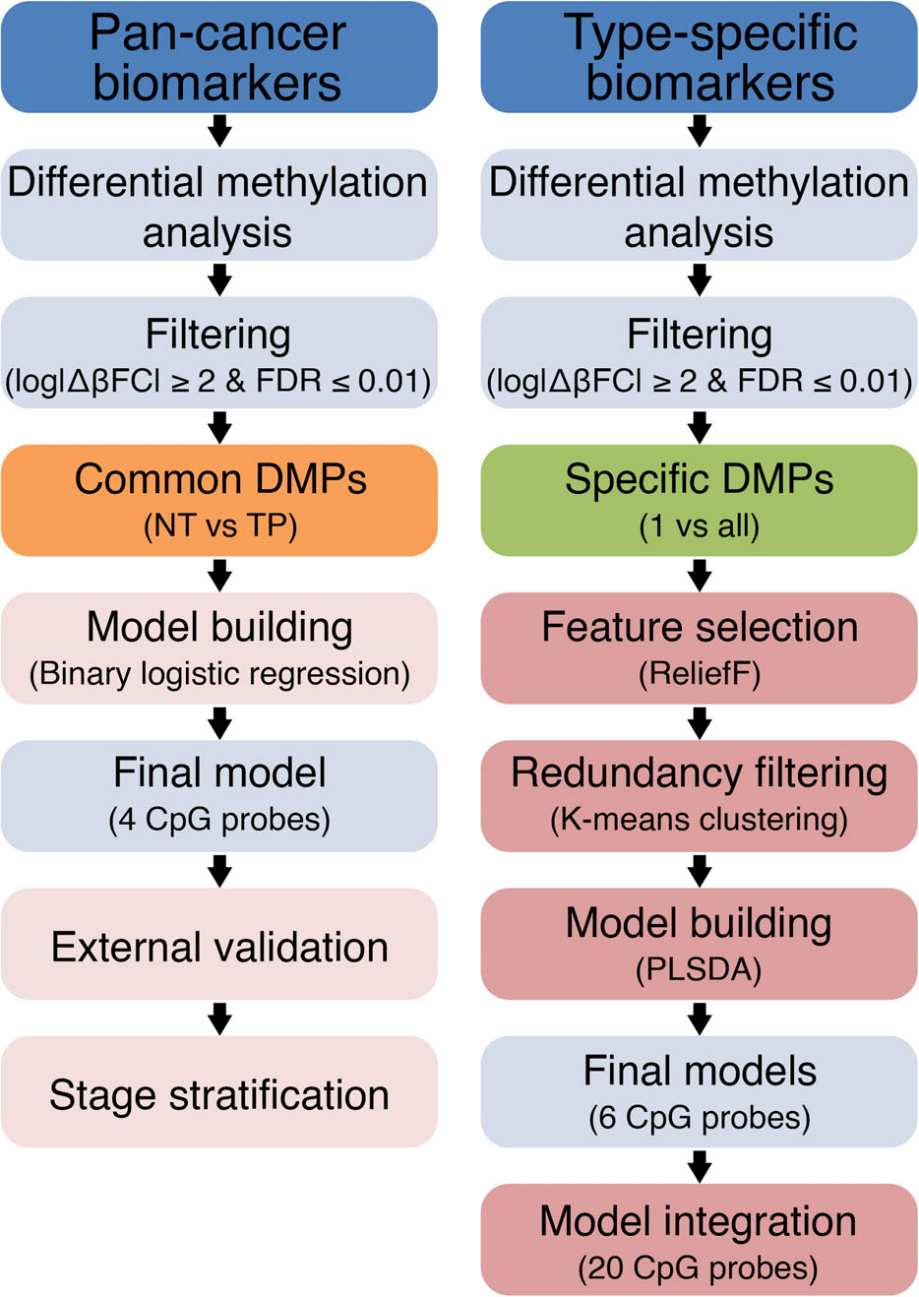
Density plot outlining the genomic distribution of differentially methylated regions across cancer types. Overall, the genomic distribution of differentially methylated regions (DMRs) looks to be similar in all of the cancer types. Highlighted regions seem to be especially conserved. A high density of DMRs can be observed in chromosomes 6, 11, and 19 in particular, while chromosome 9 seems to be completely void of any DMRs. Density is calculated and plotted in bins of 1xE6 bp.

A total of 15 260 DMRs were also identified across the tumor types with an average of 1090 DMRs per type. DMRs are extended segments of the genome (~10 bp ‐ kb) that show a quantitative alteration in DNA methylation levels across different biological samples. Similar to DMPs, kidney renal carcinoma had the most DMRs at 2505, while esophageal carcinoma had the least at 349. DMRs registered an average size of 750 bp and contained on average 12 CpG probes. An average of 726 DMRs were identified per chromosome with chromosome 6 having the most at 1962 on average and chromosome 21 the lowest at 81 on average (Table S4). DMRs had an overall similar genomic distribution across the tumor types. A segment of high DMR density on chromosome 6 seems to be present in all the tumor types. Regions with similar methylation can be seen on chromosomes 3, 11, 17, and 19. Strikingly, no DMRs could be identified on chromosome 9 in any of the cancer types (Fig. 3). A total of 29 481 DMBs were identified across the different tumors. DMBs, on the other hand, are large‐scale genomic regions (10 Kb–1 Mb) that contain hundreds of intergenic (open‐sea regions) differentially methylated CpGs [19]. On average 1785 DMBs were identified per tumor, with the most observed in kidney renal papilloma at 2543 DMBs and the least in liver carcinoma at 1020 DMBs. Being larger than DMRs and containing more CpG probes, the identified DMBs were 750 Kb long and contained 200 probes on average. 1135 DMBs could be mapped on average per chromosome, with chromosome 2 and chromosome 18 having the highest and lowest number of DMBs, respectively (Table S5). Looking at the genomic distribution of DMBs, they seem to show universal features across various cancers. This can be clearly observed in chromosomes 1, 8, 9, 18, 19, 21, and 22 (Fig. S3).

### 3.2 Methylation as a pan‐cancer detection biomarker

**Fig. 4:**
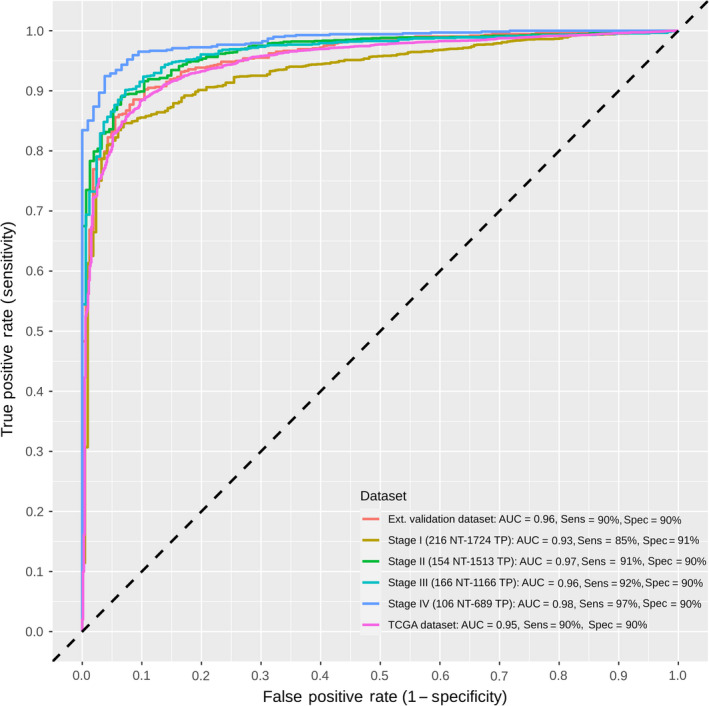
Receiver operating characteristic (ROC) curves of the final pan‐cancer model, validation datasets, and stage stratification. The final model included four CpG probes and accounted for age and tumor stage. Sensitivity and specificity at various cutoff values for the datasets and stages are plotted. The final model yielded an area under the curve (AUC) of 0.95 and a sensitivity and specificity of 90% with similar metrics when validated. Predictions in stage I were the poorest, with an AUC of 0.93 a sensitivity of 85% and a specificity of 91%. Performance in the subsequent stages improved, reaching an AUC of 0.98 and a sensitivity and specificity of 97% and 90%, respectively, in stage IV. The dotted diagonal line represents the line of no discrimination between tumor and normal tissues. AUC, area under the curve; NT, normal tissue; TP, primary tumor.

Following the filtering steps (Fig. 2), we identified 28 pan‐cancer DMPs, that were hypermethylated in the tumor samples as compared to normals across all cancer types (Fig. S2 and Tables S3 and S4). Twelve of them could be mapped to 12 distinct genes. 20 DMPs were located in CpG island regions, three in shore regions, three in open regions, and two in shelf regions (Table S6). We then used binary logistic regression to test combinations of the 28 probes that worked best in classifying samples pan‐cancer. The logistic regression classifier models were built using combinations of 1–4 probes and externally validated in the pooled GEO dataset. In total, 24 157 predictor combinations were tested of which 20 475 comprised 4 probes (Fig. S4). The average area under the curve (AUC) was 0.84 using only a single probe (Fig. S4A) and went up to 0.92 using combinations of four probes (Fig. S4D). The reported average misclassification error was 0.11 and 0.09 for 1 and 4 predictor combinations, respectively. The models performed well in the validation datasets with average external AUC for single predictors being 0.89 and 0.95 for four predictors (Figs S4A,D). Using combinations of five or more probes, we encountered large diminishing returns. Given the exponential increase in the number of combinations to be tested with more probes, this was not investigated. The final model was selected based on the highest achieved AUC and lowest misclassification error with the smallest standard deviation in these measurements across the bootstraps. The final model included four probes (Table 2) and reached cross validated AUC of 0.95 in the discovery set and an AUC of 0.96 in the validation set. The misclassification error rate was 0.06, while sensitivity and specificity were 90% and overall accuracy was 92%. A comparable performance was also achieved in the validation datasets (Fig. 4). No significant effects of clinical cancer stage or age on tissue type prediction could be measured. The stage final stratified model yielded somewhat uniform prediction results across all four stages with AUCs above 0.90. As expected, stage I exhibited the lowest metrics with a sensitivity and specificity of 85% and 91%, respectively, while the most accurate predictions were seen in stage IV with a sensitivity and specificity of 97% and 90%, respectively (Fig. 4).

**Table 2:** Overview of the Illumina CpG sites that were used in the final prediction models for both pan‐cancer and type‐specific classification. IGR, intergenomic region; TSS, transcription start site; UTR, untranslated region.

Probe ID	Chromosome	Strand	Gene	Genomic feature	Relation to CpG Island
cg17757602^a^	5	F	–	IGR	Island
cg26848718^a^	11	R	WT1	Body	Island
cg05422029^a^	6	F	–	IGR	Open Sea
cg22749589^a^	20	F	–	IGR	North Shore
cg02615833^b^	5	R	PCDH24	TSS200	Open Sea
cg26175343^b^	20	F	LRRN4	TSS200	South Shore
cg14266927^b^	14	R	BATF	TSS1500	Open Sea
cg16937769^b^	12	R	HOXC4	TSS1500	South Shore
cg24750391^b^	7	F	PON3	TSS1500	South Shore
cg23921838^b^	6	F	C6orf97	3'UTR	Open Sea
cg21710324^b^	1	F	TMEM63A	5'UTR	North Shore
cg10588135^b^	17	R	BCAS3	Body	Open Sea
cg00851394^b^	19	F	CNTD2	Body	Island
cg22966302^b^	19	F	NFIX	Body	North Shelf
cg00501869^b^	7	R	PTPRN2	Body	Island
cg23313005^b^	5	F	FAM193B	Body	Open Sea
cg12126990^b^	2	R	AFF3	Body	Open Sea
cg24686845^b^	12	F	PLXNC1	Body	Open Sea
cg10210594^b^	1	F	–	IGR	Island
cg11518509^b^	1	R	–	IGR	Open Sea
cg19251600^b^	6	F	–	IGR	Open Sea
cg16196175^b^	7	F	–	IGR	North Shore
cg21312554^b^	8	R	–	IGR	Open Sea
cg27628707^b^	15	R	–	IGR	Open Sea

^a^ Denotes pan‐cancer CpG markers.

^b^ Denotes type‐specific CPG markers.

### 3.3 Methylation as a type‐specific detection biomarker

**Fig. 5:**
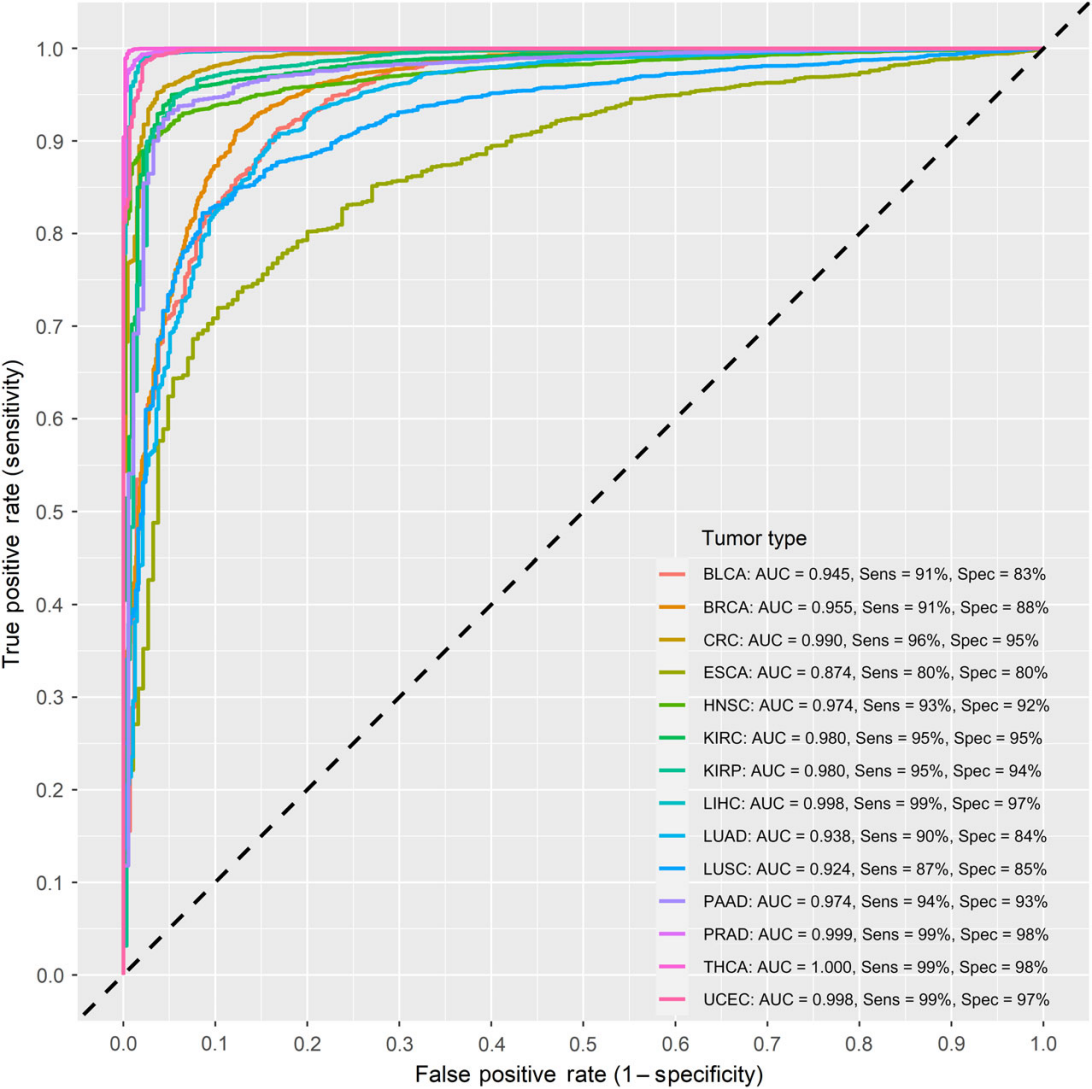
Receiver operating characteristic curves of the final integrated type‐specific partial least squares‐discriminant analysis model. The final model integrated the best performing 20 type‐specific CpG probes as predictors. The curves represent 10‐fold cross‐validated area under the curve (AUC) for classifying each of the different cancer types in a 1‐vs‐all approach. Sensitivity and specificity at various cutoff values for the datasets are plotted. The final model performed highly in classifying tumor types with all metrics above 90% for the majority of tumors. Thyroid carcinomas registered could be classified with near perfect efficiency, while esophageal carcinomas registered the lowest performance metrics with an AUC of 0.87 and both a sensitivity and specificity of 80%. The dotted diagonal line represents the line of no discrimination between tumor and normal tissues. AUC = area under the curve.

**Fig. 6:**
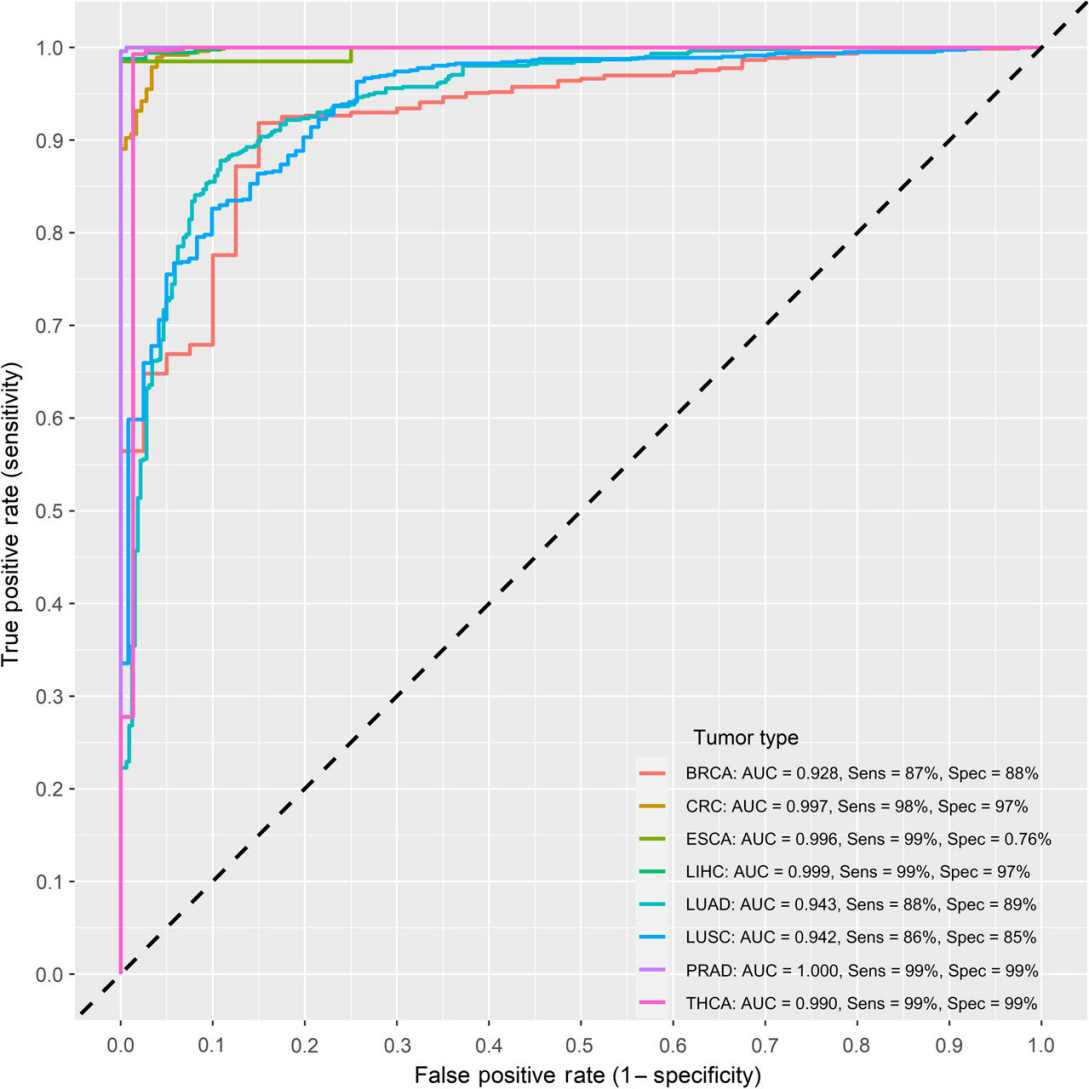
Receiver operating characteristic curves of the final integrated type‐specific partial least squares‐discriminant model in the GEO validation datasets. 11 GEO datasets were used for external validation including 332 normal and 931 tumor tissues and covering eight tumor types. The curves represent 10‐fold cross validated area under the curve (AUC) for classifying each of the different cancer types in a 1‐vs‐all approach. Sensitivity and specificity at various cutoff values for the datasets are plotted. The validated performance was very similar to the discovery dataset with most sensitivities and specificities above 90%. The dotted diagonal line represents the line of no discrimination between tumor and normal tissues. AUC = area under the curve.

After applying similar filter steps to the one‐vs‐all differential methylation analysis, colorectal cancer recorded the largest number of type‐specific DMPs at 5181, followed by thyroid cancer at 4666. The lowest number of type‐specific DMPs was recorded for esophageal cancer and lung squamous cell carcinoma at 13 and 12 DMPs, respectively (Fig. S2). After feature selection of relevant predictors, we were left with 586 probes, roughly 3% of the initial feature set. Subsequently, the redundancy filter clustering step resulted in 20 CpG predictors, 2 from each of the 10 resulting feature clusters. Combinations consisting of 6 out for these 20 predictors were used to build the classifier models; this was based on preliminary data showing the highest average AUC, with the least number of predictors, and the most practical total number of combinations to test. A total of 38 760 combinations were tested using the PLSDA algorithm using a pooled dataset of tumors across the 14 types. The mean cross‐validated AUC for classifying the 14 tumor types was 0.85. The majority of tested combinations performed well in classifying cancer types, with thyroid, urethral, prostate, kidney, colorectal, liver, and head and neck cancers having local AUC means above 0.90 (Figs S5–S7). Pancreatic, lung, esophageal, breast, and bladder cancers exhibited lower mean AUCs on average, but their local AUC maxima were all above the 0.80 mark. In fact, only esophageal cancer scored a maximal detection AUC below 0.90, at 0.87, being the most problematic to discriminate amongst the 14 types under study. Liver, prostate, uterine, and thyroid cancers could be discriminated with the highest power against all other types with an AUC of 0.99, while colorectal cancer followed with an AUC of 0.98 (Figs S5–S7). The integrated final model included 20 unique CpG probes (Table 2) and performed very well in classifying tumor types, with a measurable increase in metrics, especially specificity, as compared to individual models with 6‐probe combinations (Fig. 5 and Fig. S7). The model performed equally well in the validation set for the available cancer types (Fig. 6). Thyroid, uterine, prostate, liver, and colorectal tumors could be identified with near perfect sensitivity. Twelve of the 14 cancer types under study could be discriminated with sensitivities and specificities above 90%. Only esophageal and lung squamous cell carcinomas registered specificities at 80% and 87%, respectively (Fig. 5).

## 4 Discussion

Epigenetic alterations, such as DNA methylation, are an important regulator of gene transcription and expression. Given DNA methylation’s role in carcinogenesis and its possible use for diagnosis and therapy, our study focused on providing a comprehensive analysis of DNA methylation patterns in 14 different tumor types and identifying potential detection biomarkers. Overall, we observed that differential methylation patterns vary largely between different cancers, which is in line with other TCGA based studies involving methylation [20, 21, 22] and with the general dogma of intertumor epigenetic heterogeneity [23]. Hypomethylated CpGs constituted the larger portion of DMPs in all cancer types. This could be due to a larger portion of intergenic CpG probes on the Illumina array, where global hypomethylation in cancer is observed [24]. Gene body CpGs were the largest single portion and accounted for around 35% on average of DMPs. Currently, the exact function of gene body CpGs in cancer is not yet known. Several possible roles have been described, these include long‐range regulation, alternative promoter modulation [25] and protection against spurious transcription initiation by RNA polymerase II [26]. Demethylation of gene bodies has also been associated with gene expression in oncogenes [27]. Similar figures for these regions have also been reported in methylation array studies not involving TCGA datasets [28, 29]. CpG islands are normally located in promotor regions of genes and are typical sites of hypermethylation in cancer and constituted one of the highest portions of DMPs at around 35%, as expected. The highest proportion of DMPs was located in open‐sea regions which was also reported by Ding et al. [22] and could again be attributed to the larger portion of open‐sea probes interrogated by the Illumina array. Open‐sea regions are often hypomethylated in cancer and are associated with chromosomal instability, gene transcription, and loss of imprinting [30], all of which are characteristics of carcinogenesis. In line with similar studies [12, 31], around 21% of DMPs in our study belong to shore regions. These are known for widespread hypomethylation and contribution to cancer progression by causing chromatin instability [31].

We also investigated genome‐wide differentially methylated regions and blocks, which are thought to play a role in cancer development and progression, analogous to DMPs, as they house regulatory elements and transcription factor binding sites [32], to the best of our knowledge, this is the first time such an analysis has been reported. We observed an overall similarity in DMR/DMB number and genomic distribution across cancer types. The vast majority of identified DMBs were hypomethylated, which is to be expected as the algorithm scans only open‐sea regions where hypomethylation is widespread [17]. Despite large‐scale studies involving genome‐wide DMBs being relatively scarce, our findings align with early works suggesting that large hypomethylated blocks are a universal feature of solid tumors [30]. Hansen et al. [33] even suggested that such hypomethylated blocks could encompass half of the genome and cause extreme variability in gene expression. Several DMRs showed distinct universal features across all 14 cancer types and were hypermethylated in general. The short arm of chromosome 6 (6p) in particular exhibited a sharp DMR peak in all cancers. Interestingly, chromosome 6p is known to harbor several oncogenes that play a direct role in tumor progression. Chromosome 6 amplification has also been linked to cancer progression [34]. Moreover, chromosome 6p houses the human major histocompatibility complex (MHC). MHC class I antigen presentation is often impaired in cancer cells, which is one of the avenues by which cancer cells evade T‐cell destruction [35]. The mechanism of MHC‐I loss or downregulation in several cancers has been attributed to the hypermethylation of MHC‐I genes, suppressing their expression [36]. Therapy approaches targeting the recovery of MHC class I expression on tumor cells can be an effective form of immunotherapy, where the reversible nature of methylation plays a major role [37]. Irrespective of the specific mechanisms behind the observed patterns, DMRs and DMBs are very interesting features for further examination for diagnostic/prognostic potential in cancer. The identified regions themselves could serve as a marker site for targeted methylation sequencing assays for cancer detection, for example. They can also be used as a source of differentially methylated CpG sites that can be included in methylation marker panels. To the best of our knowledge, our work is the first large‐scale pan‐cancer analysis involving DMBs and DMRs.

Much improvement in methylation‐based cancer assays has been made in the past few years, yet most of the existing markers still target a single cancer type only, and even the best‐established ones show diagnostic shortcomings in different tumor stages [6, 38]. We used pan‐cancer differential methylation patterns which were similar in all tumors and distinct from all normal tissues to test 28 candidate CpGs for their capacity to differentiate tumors from normals. By testing out combinations of 4 CpG probes, our final model could effectively classify tumor and normal tissues with high accuracy in a pan‐cancer setting. The suggested model also performed up to par in several external validation datasets. This reinforces the model’s scalability over external data and its generalizability over a multitude of tumor types. In comparison to another TCGA‐based study that focused only on CpGs in islands and promoter regions for marker identification [39], our method focuses on identifying the most informative CpG sites irrespective of genomic location or gene. As Koch et al. point out, traditional methylation biomarker studies have focused predominantly on promoter CpG islands of tumor suppressor genes, but CpGs are not all functionally equivalent, even those within the same CpG island. The most important premise of biomarker identification is thus determining the most clinically relevant locations for accurate diagnosis [40]. Aberrant DNA methylation varies greatly in cancer based on tumor stage and current biomarkers are still underperforming in detecting early‐stage disease [41, 42]. This aspect has been absent in recent methylation‐based biomarker studies [22, 39, 43]. Our proposed CpG predictors showed no significant effect of age and tumor stage and performed equally well when stage stratified. Naturally, sensitivity and specificity were higher in the more advanced cancer stages, but the model was also able to accurately classify tumor samples even in stage I. We could not find an overlap between our marker selection and those in similar studies—nor is there an overlap between these studies themselves—[14, 39], but this is expected due to the different classifiers used in the different studies. This, however, stresses the richness of the methylome as a source of cancer markers.

Tissue‐specific methylation signatures in several cancers have already been reported [4, 44]; this forms the basis of type‐specific biomarker identification. We aimed to identify type‐specific methylation markers that could single out each cancer type from a pool of different cancers based on differential methylation in different tumors. We selected the best predictors for each cancer type and integrated those into a final 20‐CpG model that could identify 14 tumor types with high sensitivity and specificity. Recently, Liu et al. [45] showed that DNA methylation biomarkers, identified using bisulfite sequencing, could detect cancer tissue of origin in liquid biopsies. Our approach, however, yields a bigger pool from which markers could be selected; this drastically increases the number of CpG predictors and could be very beneficial particularly for liquid biopsies where circulating tumor DNA is fragmented and low in concentration. Similar to the pan‐cancer setting, probes in the final model were not limited to promoter regions; a third of them belonged to gene body regions and a quarter belonged to shore regions. This again stresses the importance of non‐promoter/island CpGs, especially in a tumor‐specific setting. Irizarry et al. [12], for example, have outlined tissue‐specific gene expression patterns associated with methylation alterations in shore regions. Several other works have also suggested that gene body regions play a pivotal role in gene expression regulation [11, 46]. Esophageal cancer showed limited detection sensitivity, which can be linked to its low differential methylation but further investigation could shed light on similarities between its methylation patterns and other cancer types. Contrastingly, the other tumors had very high sensitivities, which can prove essential in a clinical setting. The heterogeneous nature of TCGA datasets adds to the complexity of multiclass predictions. We therefore had to employ feature selection and redundancy filters to find only the most informative predictors. Moreover, a large portion of DNA methylation variability in cancer is attributed to genomic variation, albeit a major fraction may also be the result of tissue‐specific pathogenic signaling cascades [47].

The classifier models we presented exhibit high sensitivity *in silico* and follow a conservative approach for classifying tissues, while minimizing false positives. Using methylation in place of mutations for example for cancer detection holds a big technical advantage especially in liquid biopsies, as studies have shown that early‐stage patients show less mutations than the detection limit of downstream mutation assessment technologies [48, 49]. The performance of our proposed methylation markers, in both pan‐cancer and tumor‐specific settings, makes them an attractive for inclusion in a minimally invasive blood‐based diagnostic/detection assay. This can be achieved using digital droplet PCR or array technologies. Despite the recent flurry in DNA methylation *in vitro* diagnostics, to date, only a couple of methylation markers have been well established in the clinical setting, and even fewer pan‐cancer markers. Many recent strides in diagnostic and prognostic tests have been made, but current clinically available methods still target only a few cancer types or show a variable performance in detection based on tumor type and stage [7]. The IvyGene test for example uses 46 markers to identify four cancer types from cell‐free DNA (cfDNA) [50]. Our proposed model uses less information to make a wider classification; evidently, this analysis is based on tissue biopsy samples and not cfDNA, but several methylation markers have shown similar performance in both biopsy types [51]. Another current assay is the EPICUP which also employs markers from Illumina 450K methylation arrays to classify cancer samples with 87% accuracy. This test, however, at the time of writing, has not been updated with a more comprehensive list of CpG sites from the newer Infinium MethylationEPIC BeadChip array, for example [7]. We believe that a good initial selection of methylation markers that can give both pan‐cancer and cancer‐specific performance could provide the breakthrough needed for the field. By implementing feature selection and redundancy filtering steps, our computational approach for example could be used to update existing assays with highly informative CpG sites. Moreover, it can be used to swiftly identify new DMPs as potential methylation markers from new DNA methylation quantification assays such as enzyme‐based DNA conversion methods (Enzymatic Methyl‐seq [52] and TET‐assisted pyridine borane sequencing [53]), which coupled with third‐generation sequencing technologies allow for an increased recovery of amplifiable DNA over bisulfite treatment and thus an increase in the total number of profiled CpG sites. Another point that can be used for improved detection is the integration of methylation markers with existing assays that use mutation, gene expression, and/or protein expression [43]. The CancerSEEK assay has already paved the way for this by combining circulating tumor DNA (ctDNA) sequencing data with serum protein markers. Despite reporting high specificity, CancerSEEK had varying sensitivity based on cancer type but the authors advocate the use of additional markers, such as methylation to increase overall performance [49, 54]. Such assays are very promising but still have the limitation of only including patients with symptomatic cancers. Moreover, when holding specificity at 95%, their sensitivity in early stages was below average. Our analysis has showed that CpG methylation possesses significant potential as a highly informative biomarker that warrants further development and integration in novel *in vitro* cancer diagnostics.

## 5 Conclusion

Based on our previous works studying methylation markers, our approach tried to maximize classification performance with minimal predictors, especially for pan‐cancer markers. This approach provides a comprehensive reference on genome‐wide methylation patterns in several of the most common cancer types. It also highlights the epigenome as an excellent source of cancer biomarkers, which could function both as pan‐cancer and as cancer‐specific detection markers. Here, we present a selection of highly robust and informative CpG sites that can be used as effective biomarkers for cancer detection. The observed widespread changes in methylation across the genome, however, outline methylation as an important starting point for biomarker identification. Ultimately, our findings highlight DNA methylation biomarkers as encouraging avenues for the molecular characterization of cancer, through the development of minimally invasive blood‐based assays, or integration in a multi‐analyte test panel.

## Conflict of interest

The authors declare no conflict of interest.

### Peer Review

The peer review history for this article is available at https://publons.com/publon/10.1002/1878‐0261.13176.

## Author contributions

Conceptualization, GVC, KOdB, MP, and JI; methodology, GVC, KOdB, JI, EF, and MP; software, JI; validation, JI, EF, GVC, and KOdB; formal analysis, JI and EF; investigation, JI; resources, GVC, KOdB, and MP; writing‐original draft preparation, JI; writing‐review and editing, GVC, KOdB, EF, and MP; visualization, JI, supervision, GVC, KOdB, and MP; All authors read and approved the final manuscript.

## Supporting information


**Fig. S1.** Upset plot showing the number of DMPs that were common to all cancer types and those that were found in cancer types individually.
**Fig. S2.** Upset plot showing the number of pan‐cancer and tumor‐specific DMPs.
**Fig. S3.** Density plot outlining the genomic distribution of differentially methylated blocks (DMBs) across the cancer types.
**Fig. S4.** Overview of pan‐cancer model metrics for all tested predictor combinations.
**Fig. S5.** Cleveland plot overviewing the local and maximal AUC means of partial least squares‐discriminant analysis (PLSDA) models for classifying each of the 14 tumor types against all others.
**Fig. S6.** Density plot of the distribution of partial least squares‐discriminant analysis (PLSDA) cross‐validated AUCs of different 6‐probe combinations classifying each of the 14 tumor types against all others.
**Fig. S7.** Receiver operating characteristic (ROC) curves for the best performing type‐specific partial least squares‐discriminant analysis (PLSDA) models 6 CpG probes as predictors.
**Table S1.** Overview of the GEO methylation datasets used for external validation.
**Table S2.** Summary of DMPs across tumor types.

**Table S3.** Percent methylation overview of the pan‐cancer differentially methylated genes.
**Table S4.** Details of identified DMRs across tumor types.

**Table S5.** Details of identified DMBs across tumor types.

**Table S6.** Genomic details of the filtered 28 Pan‐Cancer DMPs.
**Data S1.** Detailed methods and list of used R packages.

## Data Availability

The discovery datasets analyzed during the current study are available in The Cancer Genome Atlas (TCGA) Data Portal at https://tcga‐data.nci.nih.gov. The validation datasets used during the current study are available in the Gene Expression Omnibus (GEO) database at https://www.ncbi.nlm.nih.gov/geo/ for which the individual dataset identifiers are found in Table S1 in the supplementary material.
