# Supplementary material for: Genome‐wide DNA methylation profiling and identification of potential pan‐cancer and tumor‐specific biomarkers
Source: Mol Oncol. 2022 Jan 21;16(12):2432–47. doi: 10.1002/1878-0261.13176 (PMC9208075; doi:10.1002/1878-0261.13176)
Supplement: Supplementary file 1 — Fig. S1. Upset plot showing the number of DMPs that were common to all cancer types and those that were found in cancer types individually. Fig. S2. Upset plot showing the number of pan‐cancer and tumor‐specific DMPs. Fig. S3. Density plot outlining the genomic distribution of differentially methylated blocks (DMBs) across the cancer types. Fig. S4. Overview of pan‐cancer model metrics for all tested predictor combinations. Fig. S5. Cleveland plot overviewing the local and maximal AUC means of partial least squares‐discriminant analysis (PLSDA) models for classifying each of the 14 tumor types against all others. Fig. S6. Density plot of the distribution of partial least squares‐discriminant analysis (PLSDA) cross‐validated AUCs of different 6‐probe combinations classifying each of the 14 tumor types against all others. Fig. S7. Receiver operating characteristic (ROC) curves for the best performing type‐specific partial least squares‐discriminant analysis (PLSDA) models 6 CpG probes as predictors. Table S1. Overview of the GEO methylation datasets used for external validation. Table S2. Summary of DMPs across tumor types. [file MOL2-16-2432-s002.docx]

**Supplementary Table 1. Overview of the GEO methylation datasets used for external validation.**

| GEO Dataset | #NT | #TP | #Total | Tumor Type |
| --- | --- | --- | --- | --- |
| GSE112047 | 16 | 31 | 47 | PRAD |
| GSE39279 | 0 | 306 | 306 | LUAD |
| GSE39279 | 0 | 119 | 119 | LUSC |
| GSE52826 | 8 | 4 | 12 | ESCA |
| GSE52865 | 17 | 40 | 57 | BRCA |
| GSE73549 | 15 | 57 | 72 | PRAD |
| GSE76938 | 57 | 68 | 125 | PRAD |
| GSE97466 | 50 | 91 | 141 | THCA |
| GSE89852 | 37 | 37 | 74 | LIHC |
| GSE68060 | 36 | 82 | 118 | CRC |
| GSE77718 | 96 | 96 | 192 | CRC |
| Total | 332 | 931 | 1263 | **-** |

NT = Normal tissue, TP = Primary tumor

**Supplementary Table 2. Summary of DMPs across tumor types.**

| **Tumor** | **# unique genes** | **DMPs/Gene (Max)** | **DMPs/Gene (Mode)** | **Mean DMPs/Gene** | **# DMPs with \|Δβ\| ≥ 0.3** | **# DMPs with \|Δβ\| ≥ 0.2** | **# Total DMPs** | **# Hypermethylated DMPs** | **# Hypomethylated DMPs** | **DMPs with \|Δβ\| ≥ 0.3** | **DMPs with \|Δβ\| ≥ 0.2** |
| --- | --- | --- | --- | --- | --- | --- | --- | --- | --- | --- | --- |
| **BLCA** | 17900 | 816 | 4 | 7.31 | 12577 | 44961 | 176583 | 38628 | 137955 | 7.12 | 25.46 |
| **BRCA** | 18302 | 815 | 5 | 9.49 | 4913 | 24303 | 226762 | 113507 | 113255 | 2.16 | 10.71 |
| **CRC** | 17432 | 486 | 2 | 7.18 | 11232 | 33845 | 170554 | 93449 | 77105 | 6.58 | 19.84 |
| **ESCA** | 12562 | 505 | 1 | 3.99 | 2879 | 19384 | 71046 | 31142 | 39904 | 4.05 | 27.28 |
| **HNSC** | 18269 | 788 | 4 | 8.60 | 5520 | 26444 | 208539 | 81797 | 126742 | 2.64 | 12.68 |
| **KIRC** | 18603 | 782 | 8 | 11.69 | 2012 | 10357 | 278106 | 124176 | 153930 | 0.72 | 3.72 |
| **KIRP** | 17893 | 428 | 2 | 7.19 | 2792 | 12653 | 165493 | 89891 | 75602 | 1.68 | 7.64 |
| **LIHC** | 18350 | 862 | 4 | 8.68 | 11885 | 42669 | 211873 | 52301 | 159572 | 5.60 | 20.13 |
| **LUAD** | 17290 | 702 | 1 | 6.88 | 1687 | 12432 | 162614 | 69599 | 93015 | 1.03 | 7.64 |
| **LUSC** | 18598 | 852 | 7 | 10.98 | 8311 | 32222 | 261333 | 127012 | 134321 | 3.18 | 12.32 |
| **PAAD** | 14295 | 105 | 1 | 4.28 | 1031 | 9808 | 76312 | 28598 | 47714 | 1.35 | 12.85 |
| **PRAD** | 18476 | 454 | 6 | 9.63 | 4701 | 21198 | 230314 | 121854 | 108460 | 2.04 | 9.20 |
| **THCA** | 14997 | 336 | 1 | 3.91 | 987 | 3296 | 77318 | 45156 | 32162 | 1.27 | 4.26 |
| **UCEC** | 18103 | 813 | 3 | 8.15 | 19113 | 46846 | 196696 | 125512 | 71184 | 9.71 | 23.81 |

**Supplementary Table 3. Percent methylation overview of the pan-cancer differentially methylated genes.**

Too large to fit, find attached. Suppl_Table_3_new_genes_pancancer_pecent_meth.csv

**Supplementary Table 4. Details of identified DMRs across tumor types.**

Too large to fit, find attached. Suppl_Table_5_all_DMR_info.csv

**Supplementary Table 5. Details of identified DMBs across tumor types.**

Too large to fit, find attached. Suppl_Table_6_all_DMB_info.csv

**Supplementary Table 6.** **Genomic details of the filtered 28 Pan-Cancer DMPs.**

| Name | Chromosome | Genomic Coordinate | UCSC Gene Name | Relation to CpG Island |
| --- | --- | --- | --- | --- |
| cg12071888 | 1 | 91195159 | NA | S_Shelf |
| cg18322569 | 1 | 91182777 | BARHL2 | Island |
| cg00097146 | 2 | 45171818 | SIX3 | Island |
| cg03714619 | 2 | 45160445 | NA | N_Shore |
| cg07536910 | 2 | 45171818 | EVX2 | Island |
| cg00334063 | 3 | 147106010 | ZIC4 | N_Shelf |
| cg26398467 | 3 | 147087237 | NA | NA |
| cg12353207 | 4 | 113444295 | NA | Island |
| cg17757602 | 5 | 42952113 | NA | Island |
| cg18530551 | 5 | 172670879 | NA | Island |
| cg25950112 | 5 | 170740870 | NA | N_Shore |
| cg05422029 | 6 | 27235843 | NA | NA |
| cg05452406 | 6 | 10881891 | GCM2 | Island |
| cg09513990 | 6 | 27235822 | NA | NA |
| cg16288089 | 7 | 97361408 | TAC1 | Island |
| cg21042456 | 7 | 1270558 | NA | Island |
| cg23244488 | 7 | 19146032 | NA | Island |
| cg07860213 | 8 | 70982039 | PRDM14 | Island |
| cg05667348 | 10 | 118892581 | VAX1 | Island |
| cg09537620 | 11 | 31826574 | PAX6 | Island |
| cg26848718 | 11 | 32454975 | WT1 | Island |
| cg00295794 | 13 | 100641409 | NA | Island |
| cg13692446 | 13 | 112759719 | NA | Island |
| cg24221648 | 13 | 79170283 | NA | Island |
| cg26132774 | 13 | 79170146 | NA | Island |
| cg25078444 | 14 | 29235193 | FOXG1 | Island |
| cg21200408 | 20 | 21378211 | NKX2-4 | Island |
| cg22749589 | 20 | 21501710 | NA | N_Shore |


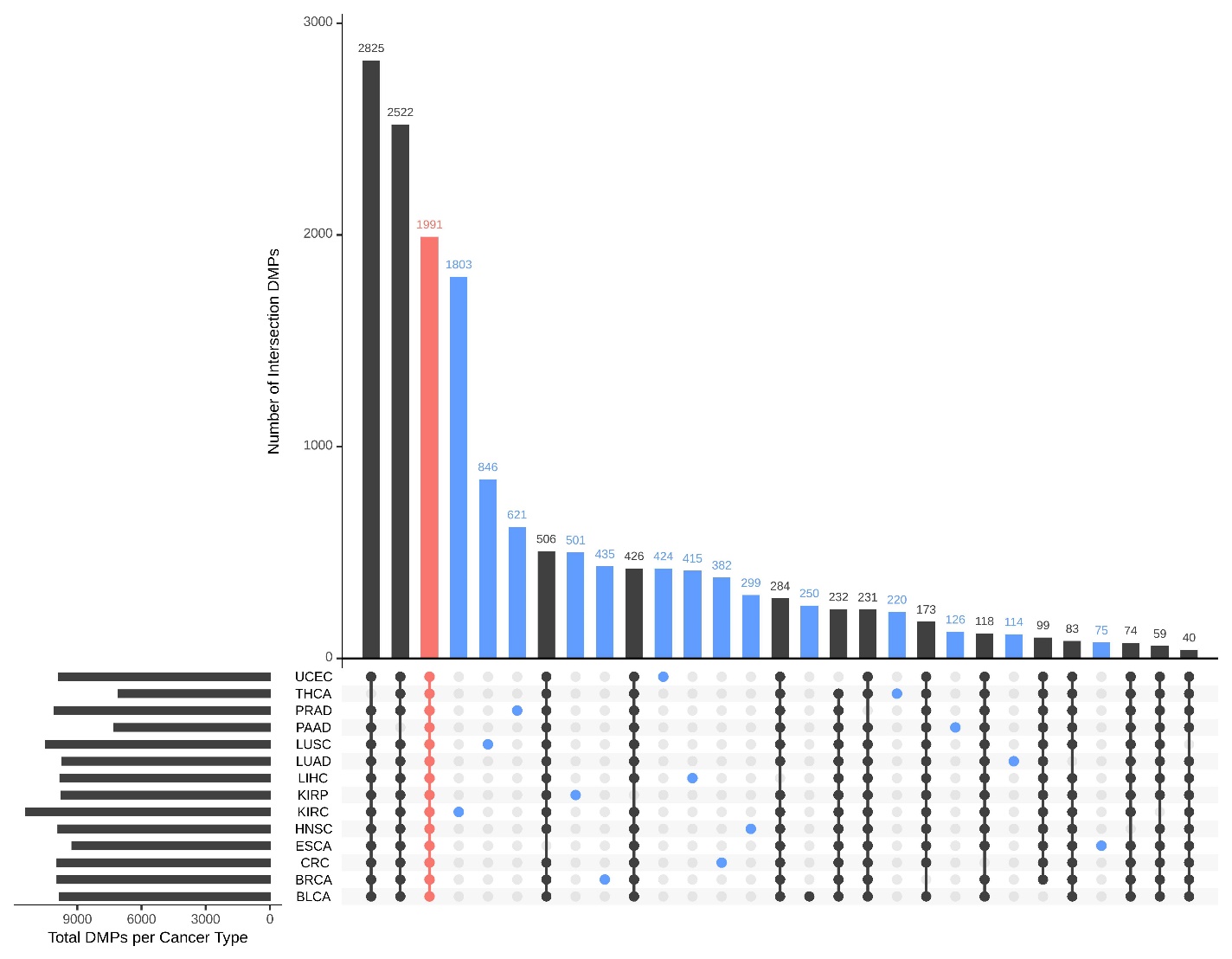


**Supplementary Figure 1. Upset plot showing the initial number of differentially methylated CpG sites that were common to all cancer types and those that were found in cancer types individually before filtering based on the set criteria.** Red dots correspond to 1991 DMPs that were common to all 14 cancer types. Blue dots correspond to DMPs that were only identified in each of the tumor types individually and not in any of the others. Black dots represent DMPs in different combinations of cancer types.


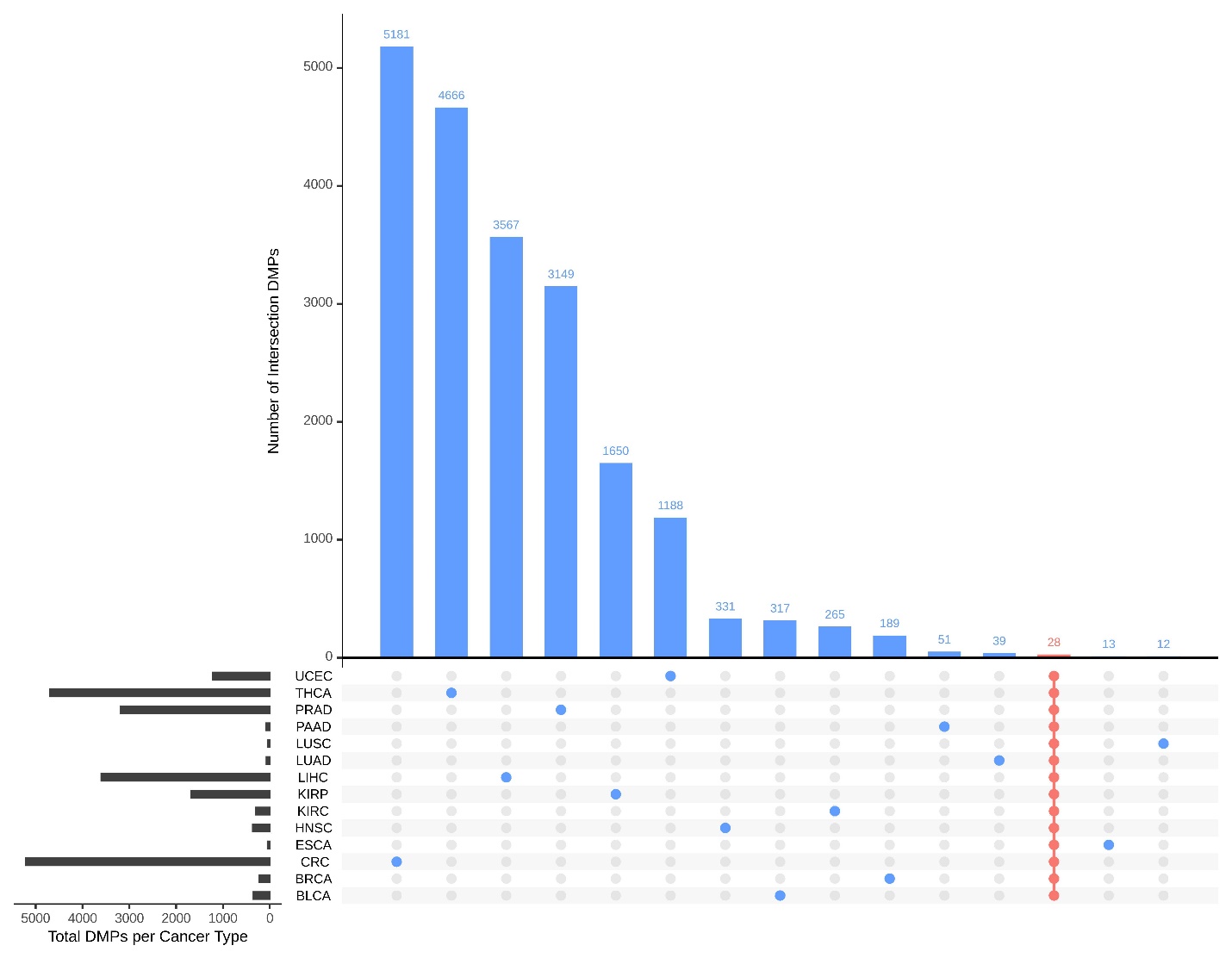


**Supplementary Figure 2. Upset plot showing the number of pan-cancer and tumor-specific DMPs that resulted after applying the set filters.** Red dots correspond to 28 DMPs that were common to all 14 cancer types. Blue dots correspond to type-specific DMPs that were only identified in each of the tumor types individually and not in any of the others. Both pan-cancer and tumor-specific DMPs were filtered from the original set of DMPs based on the following criteria: a log |ΔβFC| ≥ 2 and a p-value ≤ 0.01.


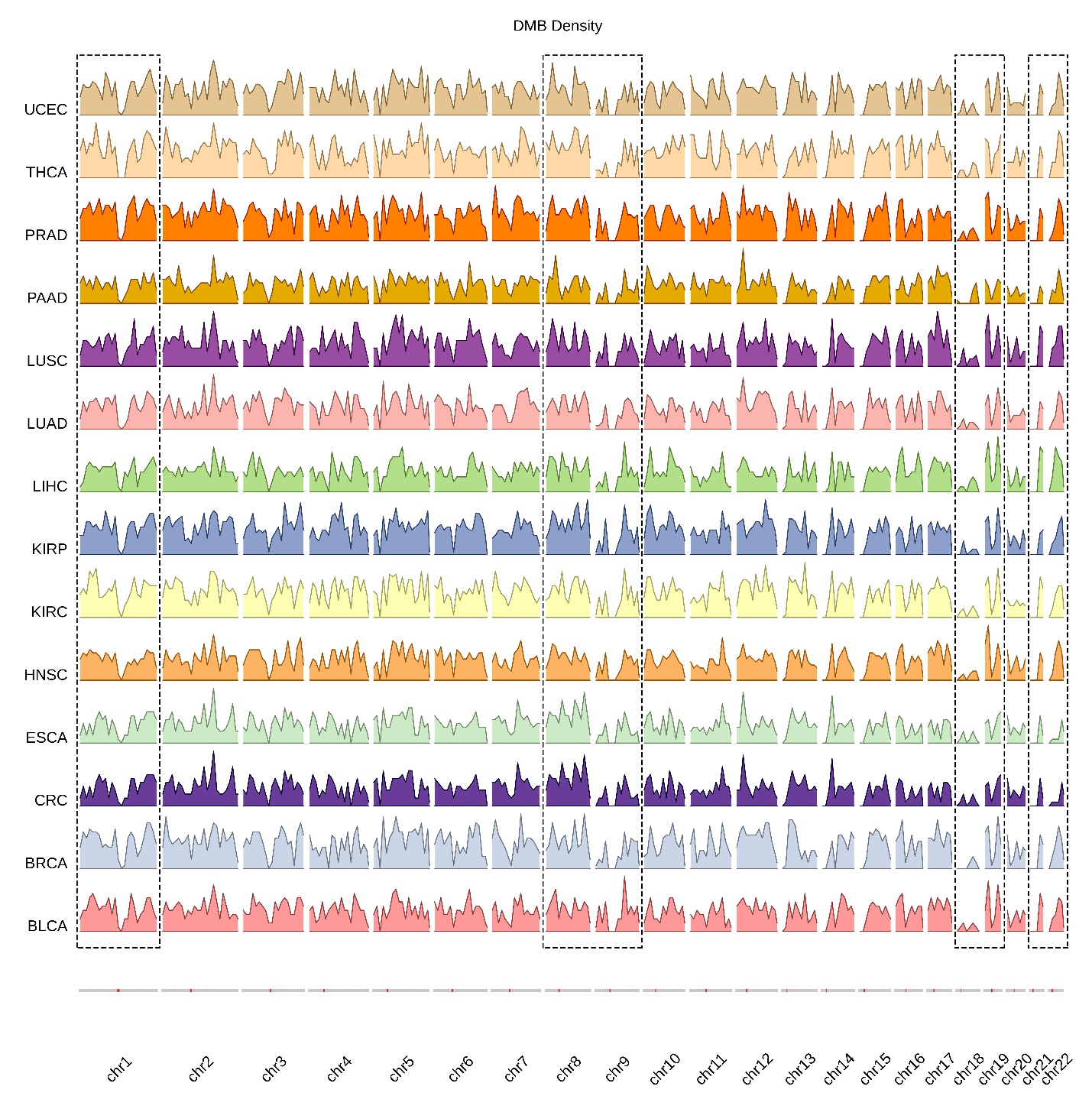


**Supplementary Figure 3. Density plot outlining the genomic distribution of differentially methylated blocks (DMBs) across the cancer types.** DMBs which are genomic segments larger than DMRs also seem to be similarly distributed across the cancer types. A high density of DMBs can be observed across the genome. Highlighted regions seem to be especially conserved, namely in chromosomes 1, 8, 9, 18, 19, 21 and 22. Density is calculated and plotted in bins of 1xE6 bp.


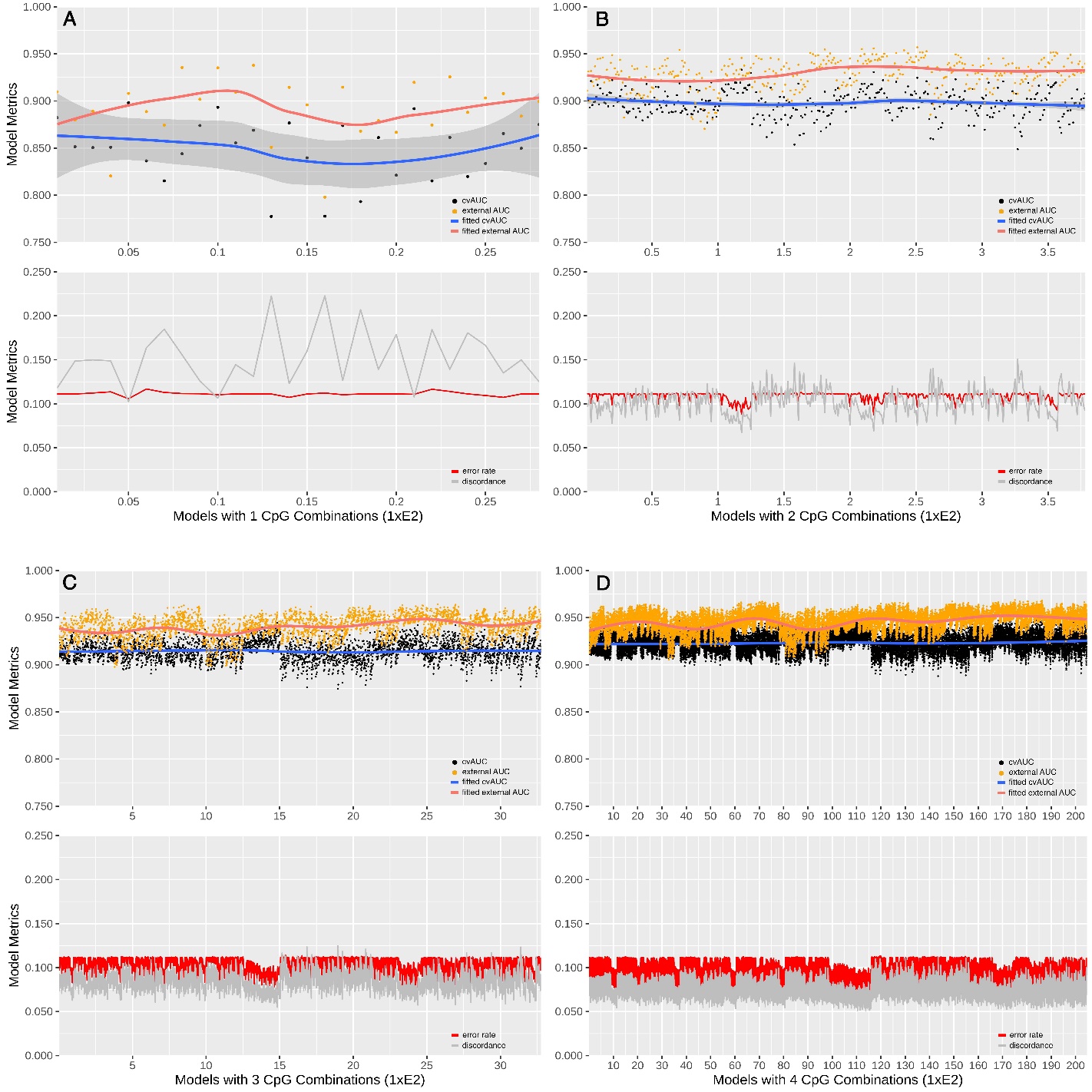


**Supplementary Figure 4. Overview of pan-cancer model metrics for all tested predictor combinations.** Each dot represents a unique predictor combination of either 1, 2, 3 or 4 CpGs sites with a total of the 24,157 tested Combinations. (A) Upper panel is a dot plot of AUC and externally-validated AUC values of models using 1 CpG as predictor. Lower panel is a line plot of discordance and error rate of models using 1 CpG as predictor. (B) Upper panel is a dot plot of AUC and externally-validated AUC values of models using 2 CpGs as predictor. Lower panel is a line plot of discordance and error rate of models using 2 CpGs as predictors. (C) Upper panel is a dot plot of AUC and externally-validated AUC values of models using 3 CpGs as predictor. Lower panel is a line plot of discordance and error rate of models using 3 CpGs as predictors. (D) Upper panel is a dot plot of AUC and externally-validated AUC values of models using 4 CpGs as predictor. Lower panel is a line plot of discordance and error rate of models using 4 CpGs as predictors. In each of the upper panels, the red and blue lines correspond to externally-validated validated AUCs and fitted AUCs respectively.


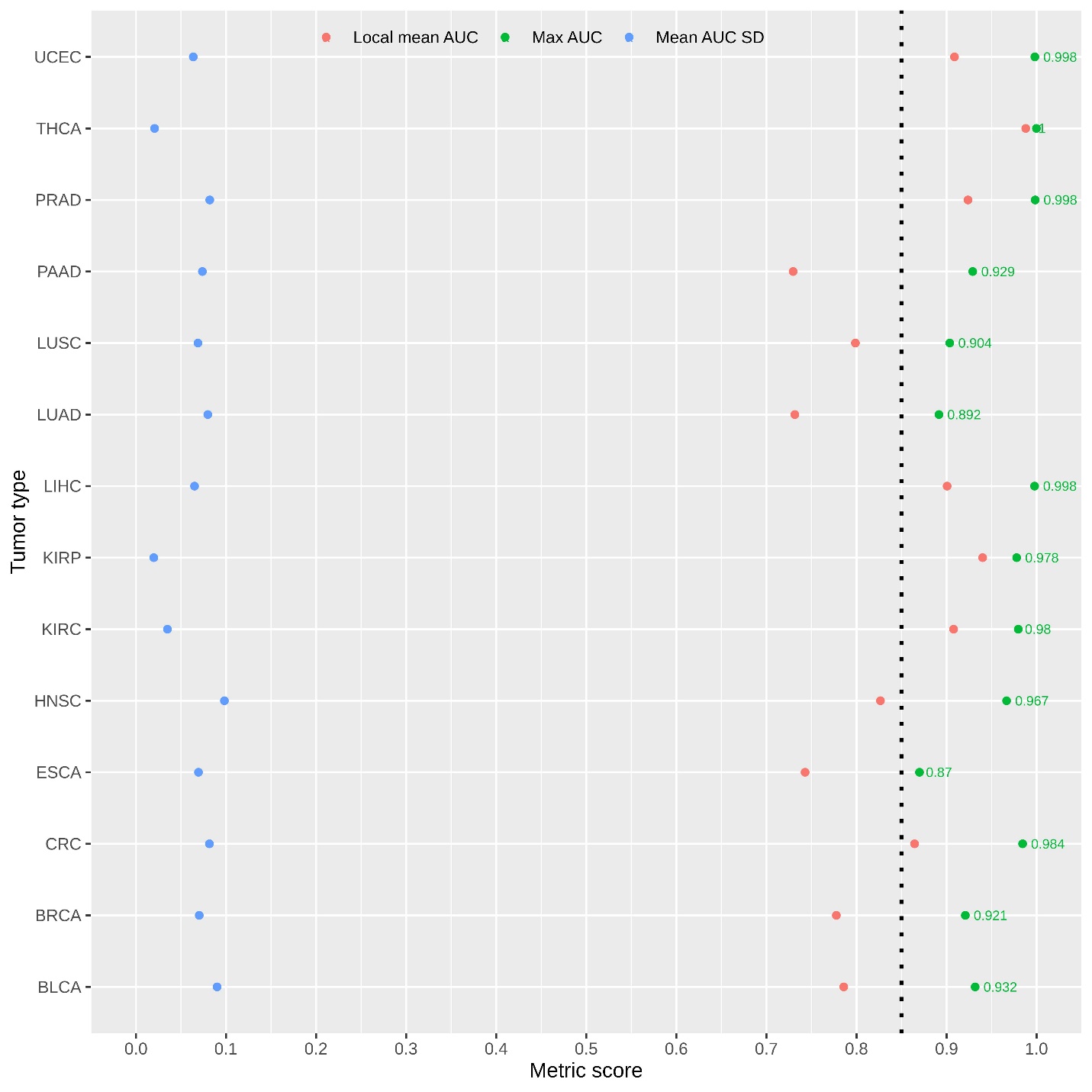
 **Supplementary Figure 5. Cleveland plot overviewing the local and maximal AUC means of partial least squares-discriminant analysis (PLSDA) models for classifying each of the 14 tumor types against all others.** The dotted line represented the overall mean AUC at 0.85. Pancreatic, lung, esophageal, breast and bladder cancers, scored lower AUC means on average, but had local AUC maxima well above the 0.80 mark. Esophageal cancer scored the lowest maximal at 0.87. Liver, prostate, uterine and thyroid cancers scored the highest maximal AUC at 0.99 while colorectal cancer followed scoring an AUC of 0.98.


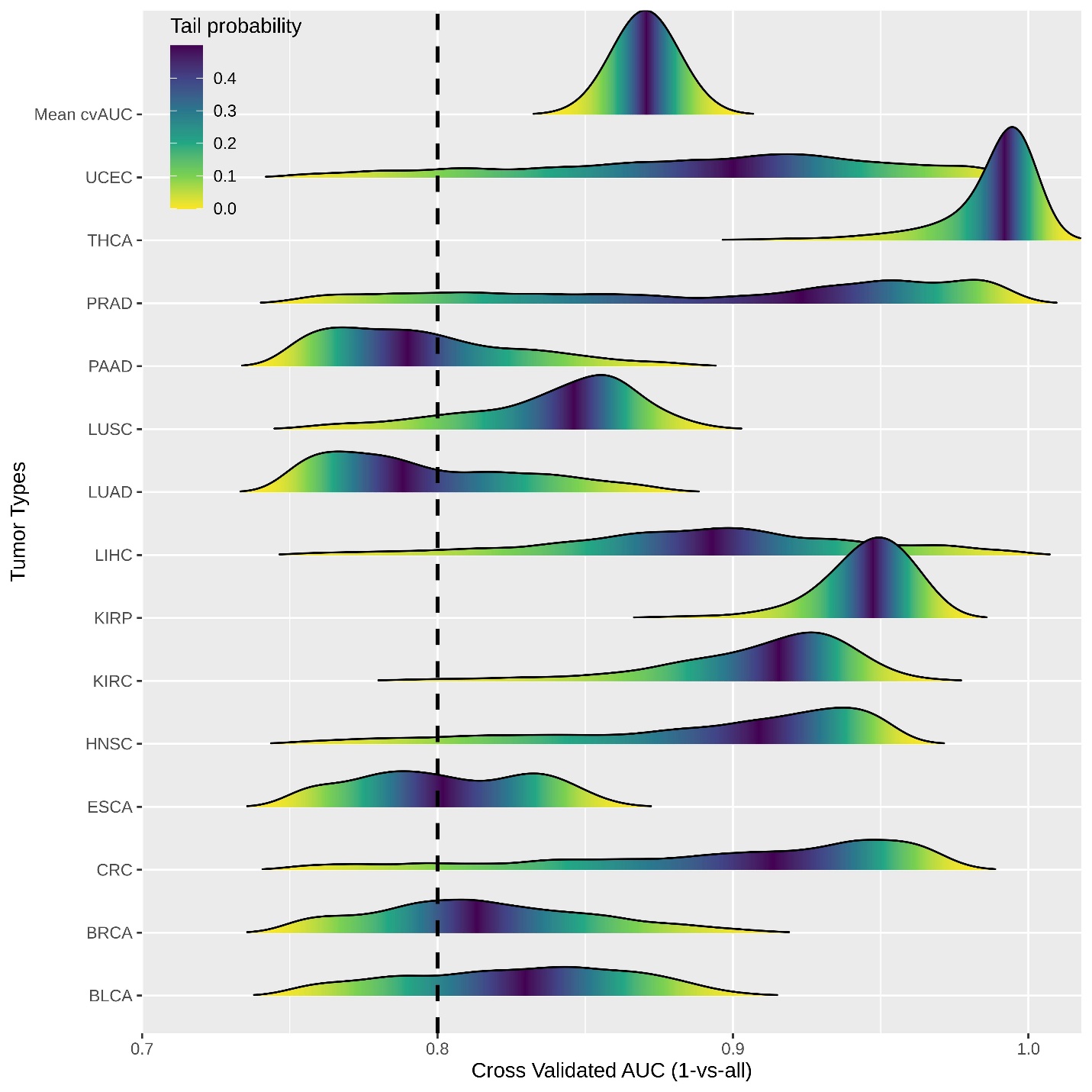


**Supplementary Figure 6. Density plot of the distribution of partial least squares-discriminant analysis (PLSDA) cross-validated AUCs of different 6-probe combinations classifying each of the 14 tumor types against all others.** A total of 38,760 6-CpG probe combinations were tested. Tail probability indicates probability that a random AUC deviates by a given amount from its expectation value. The dotted line represents the AUC 0.80 mark, which is the traditional metric for gauging the performance of classification models.


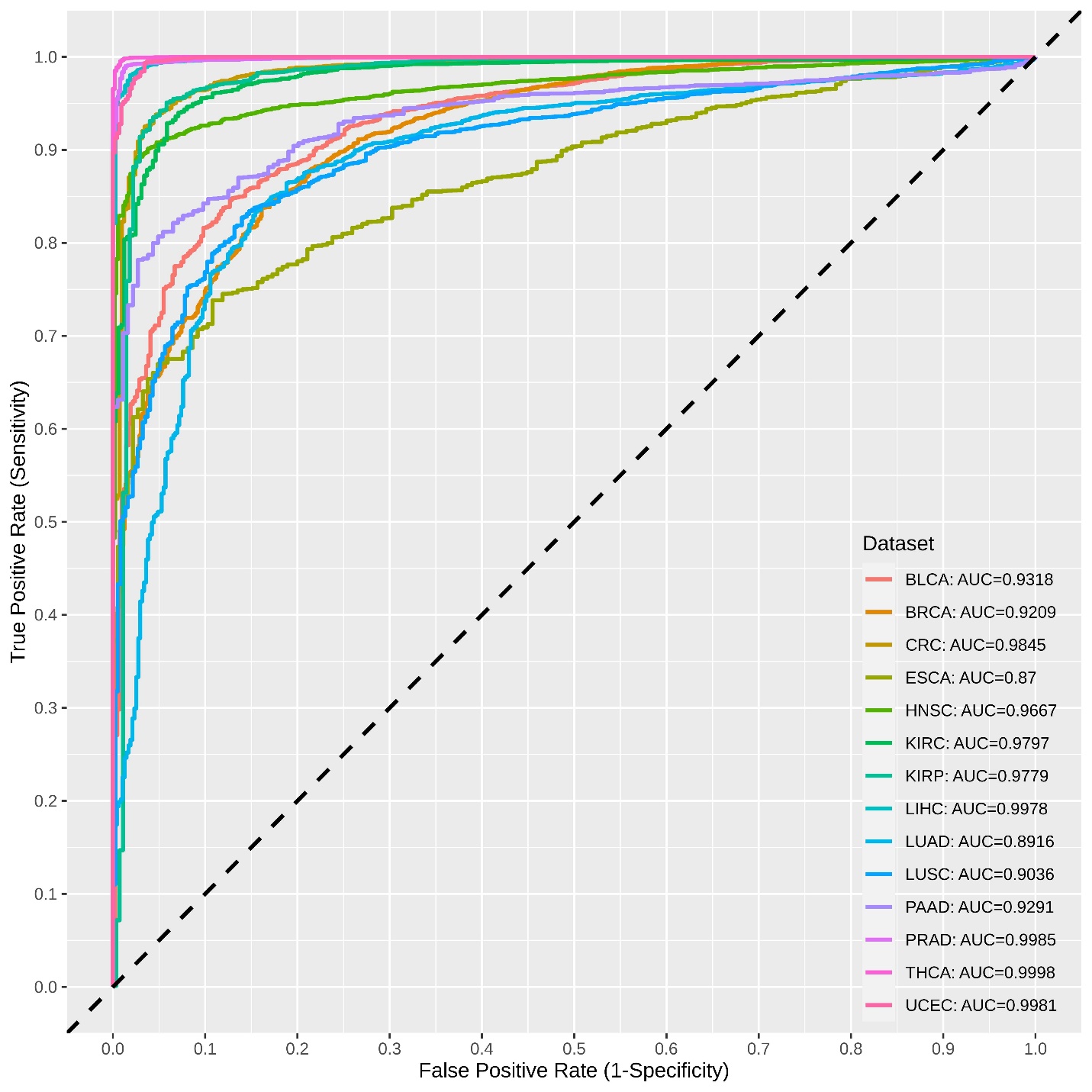


**Supplementary Figure 7. Receiver operating characteristic (ROC) curves for the best performing type-specific partial least squares-discriminant analysis (PLSDA) models 6 CpG probes as predictors.** The curves represent 10-fold cross validated AUCs for classifying each of the different cancer types in a 1-vs-all approach. Sensitivity and specificity at various cut-off values for the datasets are plotted. CpG probes included in the final integrated model comprise the combinations shown.

**Data S1**

**List of major R packages used:**

**mixOmics** - Rohart F, Gautier B, Singh A, and Le Cao K-A (2017) mixOmics: An R package for 'omics feature selection and multiple data integration. PLoS computational biology 13(11):e1005752

**lme4** - Bates D, Mächler M, Bolker B, Walker S. Fitting Linear Mixed-Effects Models using lme4. J Stat Softw. 2014;67(1):51. doi:10.18637/jss.v067.i01.

**Pbkrtest** - Halekoh U, Højsgaard S. A Kenward-Roger Approximation and Parametric Bootstrap Methods for Tests in Linear Mixed Models - The R Package pbkrtest. J Stat Softw. 2014;59(9):1-32. doi:10.18637/jss.v059.i09.

**gap** - Zhao JH. gap: Genetic Analysis Package. J Stat Softw. 2007;23(8):1-18.doi:http://dx.doi.org/10.18637/jss.v023.i08.

**MASS** - Venables WN (William N., Ripley BD, Venables WN (William N). Modern Applied Statistics With S. Vol 45. Fourth. New York: Springer; 2002. doi:10.1198/tech.2003.s33.

**ROCR** - Sing T, Sander O, Beerenwinkel N, Lengauer T. ROCR: Visualizing classifier performance in R. Bioinformatics. 2005;21(20):3940-3941. doi:10.1093/bioinformatics/bti623.

**cvAUC** - LeDell E, Petersen ML, Laan MJ van der. cvAUC: Cross-Validated Area Under the ROC Curve Confidence Intervals. 2013

**ggplot2** - H. Wickham. ggplot2: Elegant Graphics for Data Analysis. Springer-Verlag New York, 2016.

**gtools** - Gregory R. Warnes, Ben Bolker and Thomas Lumley (2020). gtools: Various R Programming Tools. R package version 3.8.2. https://CRAN.R-project.org/package=gtools

**methylGSA** - Ren, X., & Kuan, P. F. (2018). methylGSA: a Bioconductor package and Shiny app for DNA methylation data length bias adjustment in gene set testing. Bioinformatics, 35(11), 1958-1959,

**ChAMP -** Tian Y, Morris TJ, Webster AP, Yang Z, Beck S, Andrew F, Teschendorff AE (2017). “ChAMP: updated methylation analysis pipeline for Illumina BeadChips.” Bioinformatics, btx513. doi:10.1093/bioinformatics/btx513 (URL: https://doi.org/10.1093/bioinformatics/btx513).

Morris TJ, Butcher LM, Teschendorff AE, Chakravarthy AR, Wojdacz TK, Beck S (2014). “ChAMP: 450k Chip Analysis Methylation Pipeline.” _Bioinformatics_, *30*(3), 428-430. doi: 10.1093/bioinformatics/btt684 (URL:https://doi.org/10.1093/bioinformatics/btt684).

**cluster** - Maechler, M., Rousseeuw, P., Struyf, A., Hubert, M., Hornik, K.(2019). cluster: Cluster Analysis Basics and Extensions. R package version 2.1.0.

**karyoploteR** - Gel B, Serra E (2017). “karyoploteR : an R / Bioconductor package to plot customizable genomes displaying arbitrary data.” Bioinformatics, *33*(19), 3088-3090. doi: 10.1093/bioinformatics/btx346 (URL:https://doi.org/10.1093/bioinformatics/btx346).

**UpSetR** - Nils Gehlenborg (2019). UpSetR: A More Scalable Alternative to Venn and Euler Diagrams for Visualizing Intersecting Sets. R package version 1.4.0. https://CRAN.R-project.org/package=UpSetR

**ggridges** - Claus O. Wilke (2020). ggridges: Ridgeline Plots in 'ggplot2'.

**InformationValue**- Selva Prabhakaran (2016). InformationValue: Performance Analysis and Companion Functions for Binary Classification Models. R package version 1.2.3. <https://CRAN.R-project.org/package=InformationValue>
